# Dietary patterns, plasma vitamins and *Trans* fatty acids are associated with peripheral artery disease

**DOI:** 10.1186/s12944-017-0635-y

**Published:** 2017-12-28

**Authors:** Mohsen Mazidi, Nathan D. Wong, Niki Katsiki, Dimitri P. Mikhailidis, Maciej Banach

**Affiliations:** 1 Key State Laboratory of Molecular Developmental Biology, Institute of Genetics and Developmental Biology, Chinese Academy of Sciences, Chaoyang, Beijing, China; 2 Institute of Genetics and Developmental Biology. International College, University of Chinese Academy of Science (IC-UCAS), West Beichen Road, Chaoyang, China; 3 Heart Disease Prevention Program, Division of Cardiology, University of California, Irvine, CA USA; 4 Second Propedeutic Department of Internal Medicine, Medical School, Aristotle University of Thessaloniki, Hippokration Hospital, Thessaloniki, Greece; 5 Department of Clinical Biochemistry, Royal Free Campus, University College London Medical School, University College London (UCL), London, UK; 6 Department of Hypertension, Chair of Nephrology and Hypertension, Medical University of Lodz, Lodz, Poland; 7 Polish Mother’s Memorial Hospital Research Institute (PMMHRI), Lodz, Poland

**Keywords:** Peripheral artery disease, Dietary patterns, Vitamins, *Trans* fatty acids

## Abstract

**Background:**

To investigate the association between dietary patterns (DP), plasma vitamins and *trans* fatty acids (TFAs) with the likelihood of peripheral artery disease (PAD).

**Methods:**

National Health and Nutrition Examination Survey (NHANES) data for the years 1999–2002 were used. PAD was diagnosed by ankle brachial index assessment. Plasma concentrations of vitamins were measured using high performance liquid chromatography. Vitamin D levels were measured by radioimmunoassay. Analysis of covariance, principal components analysis (PCA) and adjusted logistic regression were applied, accounting for the survey design and sample weights.

**Results:**

Of the 4864 eligible participants, 2482 (51.0%) were men and 269 (5.5%) had prevalent PAD. PCA uncovered three DPs which accounted for 56.8% of the variance in dietary nutrients consumption including DP1 (fatty acids and cholesterol), DP2 (minerals, vitamins and fiber), and DP3 (polyunsaturated fatty acids [PUFA]). PAD patients had a significantly higher serum concentrations of *trans* 9-octadecenoic acid and *trans* 9, *trans* 12-octadienoic acid as well as lower plasma levels of vitamin D, retinol, retinyl stearate and retinyl palmitate (*p* < 0.001 for all comparisons). In models adjusted for age, race, diabetes, cholesterol, hypertension, smoking and energy intake, individuals in the highest quartile of the DP1 had higher odds for PAD compared with those in the lowest quartile [(odds ratio (OR): 6.43, 95% confidence interval (CI): 2.00–20.63 *p* < 0.001], while those in the highest quartile of DP2 and DP3 had lower odds of PAD relative to those in the lowest quartile (OR:0.28, OR:0.44, respectively; *p* < 0.001 for both comparisons).

**Conclusion:**

We found that quality of diet, plasma vitamins and TFAs are associated with the likelihood of PAD. If confirmed in prospective studies, the possibility that dietary factors, plasma vitamins and TFAs might be valuable for preventing or delaying the clinical progression of PAD, should be investigated in intervention trials.

**Electronic supplementary material:**

The online version of this article (10.1186/s12944-017-0635-y) contains supplementary material, which is available to authorized users.

## Background

Peripheral artery disease (PAD) is a debilitating, chronic disease caused by the development of atherosclerotic plaques in the arteries of the lower extremities. In contrast to other cardiovascular diseases, little is known about the role of diet in PAD [1]. Smoking, hypercholesterolemia, hypertension, diabetes mellitus and chronic renal failure are well-recognized risk factors for PAD besides age [2]. However, the complex relationships among these factors make it difficult to disentangle their respective contributions.

In humans, there are a few studies on PAD limited to specific nutrients, often of modest sample size [3–6]. In this regard, vitamin C has been recognised to have an anti-atherogenic effect based on the association between PAD and subclinical vitamin C deficiency [7]. Although both antioxidant and anti-inflammatory properties might explain such an effect, the possibility also exists that vitamin C deficiency is a marker of unhealthy diet or lifestyle instead of being an independent risk factor. Also omega-3 fatty acids, which are effective in both prevention and treatment of coronary artery disease, have been suggested to protect from PAD, but omega-3 fatty acid supplementation failed to improve hemodynamic or clinical outcomes in PAD patients [8]. Available studies have mostly focused on a single nutrient or food/food group in relation with PAD [3–6]. These common approaches have methodological and conceptual limitations [9, 10], in the sense that they can detect the effects of only a single nutrient or food on overall health but would not explain the interactions among nutrients and foods [11]. In addition, such studies do not provide tangible practical dietary advice, as nobody normally consumes just a single food or nutrient [10, 12].

Dietary pattern (DP) analysis has emerged as an alternative approach to nutritional epidemiology [13, 14]. In this approach, statistical methods are used to examine the pattern of intake of multiple foods or nutrients and derive single-exposure variables or DP. Such DP may provide an improved and more generalizable insight into diet-disease relations (9). The use of the DP approach could facilitate the development of public health recommendations that are clearer and more convenient to follow [15]. As people eat meals consisting of a variety of food items and complex combinations of nutrients, the traditional approach (focusing on the intake of a single micronutrient/macronutrient) does not necessarily take into account the cumulative inter-correlations and interactions between foods and nutrients [9, 16].

However, it is still largely unknown how DP may be associated with vascular disease. The purposes of this study were: (a) to identify the DP for a national sample of American adults, (b) to compare the adherence to each DP between individuals with or without PAD (at the same time correcting for main clinical and demographical confounders) for each gender, (c) to determine whether a DP analysis can have an impact on the likelihood of PAD (at the same time correcting for main clinical and demographical confounders) stratified by gender, and, (d) to determine the associations between plasma vitamins and t*rans* fatty acids (TFAs) with PAD.

## Methods

### Population characteristics

National Health and Nutrition Examination Surveys (NHANES) are ongoing repeated cross sectional surveys in non-institutionalized adults conducted by the US National Center for Health Statistics (NCHS) [17]. The National Center for Health Statistics Research Ethics Review Board approved the NHANES protocol and consent was obtained from all participants [17]. Data collection on demographic information occurs through in-home administered questionnaires, while anthropometrical, inflammation and biochemistry data are collected by trained subjects using mobile exam units. More detailed information is available elsewhere [17, 18]. Dietary intake was assessed via 24-h recall obtained by a trained interviewer during the mobile examination center visit with the use of a computer assisted dietary interview system with standardized probes, i.e. the United States department of Agriculture Automated Multiple-Pass Method (AMPM) [19–21]. AMPM is designed to enhance complete and accurate data collection while reducing respondent burden [20, 22, 23].

A blood specimen was drawn from the participant’s antecubital vein according to a standardized protocol. Fasting blood glucose (FBG) was measured in plasma by a hexokinase method using a Roche/Hitachi 911 Analyzer (DiaSorin, Stillwater, MN, USA) and Roche Modular P Chemistry Analyzer (DiaSorin, Stillwater, MN, USA)***.*** Complete laboratory procedures for collection, storage, calibration and quality control of blood samples for determination of high sensitivity C-reactive protein (hsCRP) concentrations are also available elsewhere [18, 24]. Insulin was measured using an enzyme-linked immunosorbent assay immunoassay (Mercodia, Uppsala, Sweden) [25].

The current study was based on the analysis of data for two 2-year NHANES survey cycles: 1999–2002. The ankle brachial index (ABI) assessment and the lower extremity disease examination were limited to those adults who were ≥40 years (https://www.cdc.gov/nchs/index.htm). More detailed information on the NHANES protocol is available elsewhere [17]. All methods were carried out in accordance with relevant guidelines and regulations [18, 25–27]. All experimental protocols were approved by the NCHS [18, 25–27]. Informed consent was obtained from all participants, and the NCHS research ethics review board approved the protocol.

### Plasma TFAs and vitamins concentration

Plasma TFAs were measured as part of the NHANES protocol assessing the total (free and esterified) content of selected TFAs in the plasma and providing results in concentration (μM) [28]. In this method, fatty acids in the plasma are converted into free fatty acids by subsequent acidic and alkaline hydrolysis. Free fatty acids are extracted using liquid-liquid extraction and derivatized with pentafluorobenzylbromide (PFB-Br) [28]. The derivatized fatty acids are separated by capillary gas chromatography and detected by mass spectrometry using negative chemical ionization. The fatty acids are identified based on their chromatographic retention time and the specific mass to charge ratio of the ion formed in the ion source. Retention times are then compared against those obtained with known standards [29, 30]. Quantitation is performed with standard solution using stable isotope-labelled fatty acids as internal standards. To calculate *TFAs* as percent of total fatty acids, 29 fatty acids were determined with this measurement procedure. These fatty acids comprise 95% of all fatty acids present in the plasma [30]. This method determines the following four *TFAs*: *trans*-9-hexadecenoic acid (palmitelaidic acid, C16:1n-7 t), *trans*-9-octadecenoic acid (elaidic acid, C18:1n-9 t), *trans*-11-octadecenoic acid (vaccenic acid, C18:1n-7 t-), and *trans*-9, *trans*-12-octadecadienoic acid (linolelaidic acid, C18:2n-6 t, 9 t) [28]. More detailed information on the NHANES protocol is available in the NHANES manual (https://www.cdc.gov/nchs/index.htm). Plasma concentrations of vitamins A (retinol) and two retinyl esters, were also measured using high performance liquid chromatography with photodiode array detection as previously detailed [17]. Total plasma 25(hydroxy)vitamin D was measured at the National Center for Environmental Health, CDC, Atlanta, GA, USA using a radioimmunoassay (RIA) kit (DiaSorin, Stillwater, MN, USA). The sensitivity of this assay has been shown to be 1.5 ng/ml and the coefficient of variance (CV) was 7% [31].

### PAD assessment

ABI was calculated for each participant as follows: with the participant in the supine position, trained health staff used an 8.1-MHz Doppler probe to perform the examination following a standard operation protocol. The ABI was calculated by dividing the ankle mean systolic blood pressure by brachial mean systolic blood pressure in the same side. The presence of PAD was defined as an ABI < 0.9 in either side [32]. We excluded subjects (*n* = 40) with extremely high ABI (>1.4).

### Statistical analysis

We conducted the analyses according to the guidelines by the Centers for Disease Control and Prevention (CDC) for analysis of complex NHANES datasets, accounting for the masked variance and using the proposed weighting methodology [23, 33]. Principal components analysis (PCA) was applied in order to identify DP [21, 34]. Briefly, PCA with orthogonal transformation (varimax procedure) was performed to derive nutrient patterns based on the nutrients. DPs were retained for further analysis based on their natural interpretation and eigenvalues on the Scree test [34, 35]. We computed the score for each DP by summing up intakes of nutrients weighted by their factor loadings [34, 35]. Each participant received a score for each identified DP. As simple linear dose–response relationships are unlikely to be found in nutritional epidemiology [21, 34, 36]. Analysis of covariance (ANCOVA) was applied to determine the adjusted mean of the nutrients across the quartile of the DPs and also the adjusted mean of each DP score for individuals with and without PAD stratified by gender. Adjusted logistic and linear (500 bootstrap replications) regression was used to determine the odds of PAD across the each PD and severity of PAD and adhesion to each DPs. All tests were two sided, and *p* < 0.05 was the level of significance.

## Results

A total of 4864 participants were eligible for inclusion in the current analysis. Of these, 269 (5.5%) had PAD. The characteristics of participants overall and by PAD presence are summarised in Additional file 1: Table S1. Overall 2482 (51.0%) participants were men and 2382 (49.0%) were women, with no significant difference by PAD status (*p* = 0.423). PAD patients, compared with those without PAD, comprised more of non-Hispanic Whites (57.2 vs. 52.4%), and non-Hispanic Blacks (23.4 vs. 17.6%), and less of Mexican-Americans (15.2 vs. 22.6%), other Hispanic (3.7 vs. 4.5%), or other ethnicities (0.4 vs. 2.9%) (*p* < 0.001 for differences in the distribution of ethnicity by PAD presence).

The mean age was 59.6 years overall, and it was higher in participants with PAD than in those without (70.1 vs. 58.8 years, *p* < 0.001). Furthermore, PAD patients had higher body mass index (*p* < 0.001) as well as higher serum concentrations of hsCRP, triglyceride (TG), FBG, insulin and HbA1c (*p* < 0.001 for all comparisons). Overall, only 0.8% of the study population (*n* = 40) had extremely high ABI (>1.4) and were not included in our analyses.

By applying PCA, we found three DP which could explain 56.8% of the variance of the dietary nutrients consumption. Additional file 2: Table S2 illustrates the nutrients that contributed to each DP. Accordingly, the first DP is mainly representative of saturated, total fat, cholesterol and mono-unsaturated fatty acids (MUFA), the second DP represents minerals, vitamins and fiber, and the third DP is mainly representative of polyunsaturated fatty acids (PUFA). Additional file 2: Table S2 shows the age, gender, and race-adjusted mean of each nutrient by quartiles of the three DP.

Adjusted (by age, race, diabetes, hypertension, cholesterol, smoking and energy intake) mean of score for DP2 and DP3 were higher in men without PAD (*p* < 0.001)., i.e. men without PAD had a better adherence to the diet with higher intake of minerals, vitamins and fiber (5685.0 vs. 6933.2, *p* < 0.001, Table 1) and to the diet with higher consumption of PUFA (10.8 vs. 13.9, *p* < 0.001) compared with men with PAD. We did not find any difference for adjusted mean of each DP between women (all *p* > 0.05, Table 1).

**Table 1:** Adjusted mean of dietary pattern score in patients with and without peripheral artery disease (PAD)

		Participants		*P*-value
		With PAD	Without PAD	
Male	First Dietary Pattern (Fatty Acids)	340.1 ± 17.5	358.0 ± 36.3	0.525
	Second Dietary Pattern (Minerals And Vitamins)	5679.8 ± 221.9	6959.2 ± 441.4	< 0.001
	Third Dietary Pattern (Poly Unsaturated Fatty Acid)	11.2 ± 0.6	14.2 ± 1.2	< 0.001
Female	First Dietary Pattern (Fatty Acids)	228 ± 4.9	252 ± 22.33	0.125
	Second Dietary Pattern (Minerals And Vitamins)	4603 ± 223.6	4758 ± 125.6	0.718
	Third Dietary Pattern (Poly Unsaturated Fatty Acid)	10.3 ± 0.4	10.5 ± 1.4	0.856

Analysis of covariance applied to calculate age, race, diabetes, hypertension, cholesterol, smoking and energy intake adjusted mean of dietary score. Value expressed as mean ± SEM

Furthermore, we computed adjusted (by age, race, diabetes, hypertension, cholesterol, smoking and energy intake) odds for PAD for each DP stratified by gender. The results displayed that men in the highest quartile of the DP1 had a higher odds of PAD compared with the lowest quartile [odds ratio (OR): 6.32, 95% confidence interval (95%CI): 1.89–19.25, *p* < 0.001], while men in the fourth quartile of DP2 and DP3 had lower odds for PAD compared with the first quartile (OR:0.28, 95%CI: 0.09–0.83, and OR: 0.44, 95%CI: 0.20–0.96, *p* < 0.001, respectively). In women, subjects in the fourth quartile of DP2 had 81.1% lower likelihood for PAD compared with those in the first quartile (OR: 1.66, 95%CI: 0.03–0.76, *p* < 0.001). To determine the association between PAD severity (as assessed by ABI value) and adhesion to each DP, adjusted (by age, race, diabetes, hypertension, cholesterol, smoking and energy intake) linear regression (with 500 bootstrap replications) was performed, reporting positive and significant associations between lower severity of PAD (higher ABI score) and higher score of DP2 and DP3 (for DP1 = β coefficient: 0.046, 95%CI: 0.049–0.171; for DP2 = β coefficient: 0.049, 95%CI: 0.051–0.182, *p* < 0.001 for both comparison).

When plasma TFAs and vitamins were adjusted for age, race, diabetes, hypertension, cholesterol, and smoking, PAD patients had significantly higher plasma concentrations of *trans* 9-octadecenoic acid and *trans* 9, *trans* 12-octadienoic acid and lower levels of vitamin D, retinol, retinyl stearate and retinyl palmitate (*p* < 0.001 for all comparisons**,** Table 2).

**Table 2:** Adjusted mean of *trans* fatty acids and plasma antioxidant levels in patients with and without peripheral artery disease (PAD)

	Participants		*P*-value
	With PAD	Without PAD	
Trans 9-hexadecenoic acid(uM)	6.5 ± 0.12	6.1 ± 0.19	0.842
Trans 11-octadecenoic acid (uM)	38.1 ± 0.36	34.1 ± 0.92	0.265
Trans 9-octadecenoic acid (uM)	35.2 ± 0.35	30.6 ± 0.62	< 0.001
Trans 9, trans 12-octadienoic acid (uM)	2.8 ± 0.12	2.4 ± 0.31	< 0.001
Vitamin D (nmol/L)	54.3 ± 0.28	57.1 ± 0.72	< 0.001
Retinol(μg/dL)	60.2 ± 0.62	68.4 ± 0.64	< 0.001
Retinyl stearate(μg/dL)	0.43 ± 0.01	0.87 ± 0.01	< 0.001
Retinyl palmitate(μg/dL)	2.1 ± 0.28	3.0 ± 0.10	< 0.001

Analysis of covariance applied to calculate age, race, diabetes, hypertension, cholesterol, smoking and energy intake adjusted mean of dietary score. Value expressed as mean ± SEM

## Discussion

Our findings revealed that individuals consuming a diet with a high intake of minerals, vitamins, fiber and [second DP (DP2)] PUFA [third DP (DP3)] had a lower likelihood of PAD, while diet rich in fatty acids [first DP (DP1)]) are associated with an increased likelihood of PAD. Furthermore, individuals without PAD had higher plasma vitamins concentrations and lower plasma TFAs levels compared with PAD patients.

In line with our study, previous investigators showed that PAD diagnosis and the presence of claudication correlated with a diet rich in saturated fat, sodium, and cholesterol, and low in fiber, vitamin E, folate intake and lower levels of omega-3 PUFA [37–39]. In this regard, in a small, case–control study in an Edinburgh population, docosapentaenoic acid (PUFA) was found to be protective for PAD [6]. In patients with a history of myocardial infarction, α-linoleic acids (ALA) as a key component of the Mediterranean diet, was shown to reduce the risk of cardiovascular events in one trial [40]. In a prospective study by Schiano et al., supplementing PAD patients with 2 g of omega-3 PUFA daily for 12 weeks led to an improvement in endothelial function relative to the non-supplemented control group, hinting at a possible slower progression of PAD [41]***.*** With regard to potential mechanisms, it could be suggested that PUFA were shown to decrease blood triacylglycerol concentrations, inhibit the production of chemoattractants, growth factors, adhesion molecules, inflammatory eicosanoids and cytokines [38], lower blood pressure, protect against endothelial damage and atherosclerosis [39], enhance nitrite oxide production, endothelial relaxation and vascular compliance, inhibit thrombosis and cardiac arrhythmias as well as improve heart rate variability [42]. These mechanisms most probably explain the primary and secondary CVD protection afforded by PUFA consumption, suggesting that PUFA consumption may also be beneficial in PAD. In this context, the impact of PUFA on the resolution of inflammation may eventually prove to be beneficial for patients with PAD, who have a high inflammatory burden [42].

In the present study, participants with PAD had higher levels of the *trans* 9-octadecenoic acid and *trans* 9, *trans* 12-octadienoic acid compared with PAD-free subjects which is in line with other studies [43, 44]. Previous studies have reported that TFAs may induce endothelial dysfunction and that this may be related to an upregulation of pro-inflammatory molecules, linking vascular inflammation and thrombosis [45–47, 49]. We have recently reported the potential role of TFAs intake on inflammation in humans [48–50]. These findings are in line with the studies in animals and in vitro [51], with suggestion that in TFA-exposed blood vessels, inflammation and oxidative stress may trigger pro-thrombogenic activity of endothelial cells, which then exceeds the anti-thrombogenic activity [51]. Overall, TFAs may act as a risk factor of the development of PAD via their role on inflammation.

The present investigation showed that PAD patients had lower levels of vitamins. Consistently, a nationally representative cross-sectional study of adults, intakes of various nutrients, including folate and vitamins A, C, and E were associated with a decreased prevalence of PAD in uncontrolled and partially controlled models [52]. Moreover, it has been demonstrated that PAD was associated with a lower consumption of vegetables, fiber, vitamins C and E and folate as compared with individuals without PAD [37]. The Atherosclerosis Risk In Communities (ARIC) study reported that low 25 (OH) D was associated with 30% increased risk of PAD in black and white participants [53]. Moreover, in another cross-sectional analysis researchers found an 80% higher prevalence of PAD in participants in the lowest quartile of 25(OH) D compared to the highest quartile [54].

From our PCA, we have found that subjects with higher load of fiber in diet had a lower likelihood of PAD; fiber may be beneficial in reducing inflammation, as there is a strong relationship between increased fiber intake and lower hsCRP levels [55]. Furthermore, dietary fiber may have a protective effect against developing PAD, as a positive association between cereal fiber and ABI was found in the Edinburgh Artery study [4] and the Health Professional’s Follow-up study [56]. Therefore, individuals with PAD and claudication may experience cardiovascular benefits by increasing dietary fiber intake, possibly via reduced inflammation and improved macro- and microvascular function.

Several mechanisms of action, have been suggested to explain the potential protective effect of fibers in PAD. Briefly, it has been hypothesized that the apparent beneficial effect of fiber intake against cardiovascular disease is mediated by cholesterol-lowering as well as reduced plasminogen activator inhibitor type 1 and factor VII activity, especially due to soluble fiber [57]. Elevated cytosolic triacylglycerols are associated with oxidative stress and can cause endothelial dysfunction [57], thus possibly predisposing to PAD development. Other mechanisms by which fiber intake might protect against PAD are: [1] increased insulin sensitivity, as fiber consumption improves insulin sensitivity by slowing the absorption of nutrients from the gut [58]; [2] reduced serum glucose levels [58]; [3] production of short chain fatty acids by gut bacteria consequently improving glucose metabolism [59], which is associated with lower LDL, blood pressure and triacylglycerols, and higher HDL [59]. Furthermore, among US adults (*n* = 18,741, mean age = 44.7 years) and after adjusting for age, sex, race, education, smoking, physical activity, BMI and total energy consumed, we observed a link between CRP, an independent risk factor for cardiovascular disease, and dietary pattern rich in fiber (unpublished data), indicating an effect of dietary fiber on the risk of cardiovascular disease mediated in part by inflammatory pathwa*ys.*

The current study has several strengths. To our knowledge, it is the largest study of the association of peripheral arterial disease and plasma TFAs, antioxidants, dietary pattern with PCA methods, among a representative sample of non-institutionalized US adults. Due to a large sample size, this study is sufficiently powered to test the associations. The participant selection was based on random sampling of the general population, and therefore the results obtained from nationally representative samples are generalizable to the US population. Furthermore, detailed ABI assessments were conducted with a number of quality control procedures, including calibration of techniques prior to and annually throughout the survey.

The findings of our study should be considered in the context of some limitations including those inherent to cross-sectional studies such as exposure-outcome temporal relationship identification and reverse causality. Moreover, we excluded 40 subjects with very high ABI (>1.4) due to noncompressible arteries who are technically abnormal; such individuals should be evaluated separately in other studies with larger samples. There is also a need to assess how several factors such as ethnicity, smoker status and other characteritics can influence the intake of the nutrients considered in the present study. We need also mention that our assays sensivity for measuring Vitamin D was low.

This study has important clinical and public health implications. PAD is a chronic inflammatory condition that is associated with longstanding poor nutrition and other lifestyle habits. Understanding the interplay between vascular health status, diet and plasma levels of dietary factors (TFAs, antioxidants) is an essential and crucial step concerning any use of the findings for public health strategy and preventive actions. Future studies are needed to evaluate whether PAD patients may experience clinical benefits from improved dietary habits including regular intake of fish or daily fish oil supplements, increased dietary soluble fiber intake, consumption of adequate amounts of vitamins, and minerals, as well as reduced TFAs.

## Conclusion

In conclusion, our results provide information on the association between plasma vitamins and TFAs and the likelihood of PAD. We have found that dietary factors (either via consumption or plasma concentration) are independently associated with the likelihood of PAD. The impact of the dietary items on preventing or delaying the clinical progression of PAD should be investigated in intervention trials.

## Additional files

Additional file 1: Table S1. Demographic, anthropometric and clinical characteristics of participants. (DOCX 13 kb)

Additional file 2: Table S2. Age, Gender, and Race Adjusted Mean of Nutrient Intakes Across Quartiles of Each Dietary Pattern. (DOCX 22 kb)
