# Supplementary material for: Dietary patterns, plasma vitamins and Trans fatty acids are associated with peripheral artery disease
Source: Lipids Health Dis. 2017 Dec 28;16:254. doi: 10.1186/s12944-017-0635-y (PMC5745924; doi:10.1186/s12944-017-0635-y)
Supplement: Supplementary file 1 — Demographic, anthropometric and clinical characteristics of participants. (DOCX 13 kb) [file 12944_2017_635_MOESM1_ESM.docx]

| **Table S1.** Demographic, anthropometric and clinical characteristics of participants | | | | | |
| --- | --- | --- | --- | --- | --- |
| **Characteristics** | | **Overall (n=4864)** | **With PAD (n=269)** | **Without PAD (n=4594)** | **p-value** |
| **Gender** | **Men (%)** | 51.0 | 51.7 | 51.0 | **<0.001** |
|  | **Women (%)** | 49.0 | 48.3 | 49.0 |  |
| **Age (Years), [ mean ± SEM ]** | | 59.6±0.18 | 70.1±0.21 | 58.8±0.68 | **<0.001** |
| **Race/**  **Ethnicity** | **White (non-Hispanic) (%)** | 52.7 | 57.2 | 52.4 | **<0.001** |
|  | **Non-Hispanic Black (%)** | 17.9 | 23.4 | 17.6 |  |
|  | **Mexican-American (%)** | 22.2 | 15.2 | 22.6 |  |
|  | **Other Hispanic (%)** | 4.5 | 3.7 | 4.5 |  |
|  | **Other (%)** | 2.8 | 0.4 | 2.9 |  |
| **Body mass index (kg/m^2^)** | | 28.3±0.08 | 27.4±0.09 | 24.2±0.010 | **<0.001** |
| **Serum triglycerides (mg/dl)** | | 164.8±3.2 | 165.5±3.9 | 158.2±2.9 | **<0.001** |
| **Serum total cholesterol(mg/dl)** | | 210.3±0.6 | 212.2±0.7 | 210.2±0.6 | **0.235** |
| **Serum high density lipoprotein (mg/dl)** | | 51.8±0.2 | 52.2±0.1 | 51.8±0.1 | **0.154** |
| **Serum hsCRP (mg/dl)** | | 0.48±0.01 | 0.73±0.02 | 0.47±0.01 | **<0.001** |
| **Systolic blood pressure (mmHg)** | | 130.6±0.3 | 148.2±0.2 | 130.2±0.2 | **<0.001** |
| **Diastolic blood pressure (mmHg)** | | 73.2±0.2 | 68.6±0.2 | 74.1±0.1 | **0.125** |
| **Fasting blood glucose (mg/dl)** | | 109.2±0.7 | 120.6±0.6 | 108.8±0.7 | **<0.001** |
| **Plasma insulin (μU/mL)** | | 14.5±0.4 | 21.0±0.3 | 14.2±0.4 | **<0.001** |
| **HbA1c (%)** | | 5.7±0.01 | 6.2±0.01 | 5.7±0.02 | **<0.001** |
| ***PAD: Peripheral artery disease, hsCRP: High-sensitivity C-reactive Protein, HbA1c: Hemoglobin A1c. Values expressed as mean ≥SEM or percent.*** | | | | | |
