# Supplementary material for: Dietary patterns, plasma vitamins and Trans fatty acids are associated with peripheral artery disease
Source: Lipids Health Dis. 2017 Dec 28;16:254. doi: 10.1186/s12944-017-0635-y (PMC5745924; doi:10.1186/s12944-017-0635-y)
Supplement: Supplementary file 2 — Age, Gender, and Race Adjusted Mean of Nutrient Intakes Across Quartiles of Each Dietary Pattern. (DOCX 22 kb) [file 12944_2017_635_MOESM2_ESM.docx]

| **Table S2:** Age, Gender, and Race Adjusted Mean of Nutrient Intakes Across Quartiles of Each Dietary Pattern | | | | | | | | | | | | | |
| --- | --- | --- | --- | --- | --- | --- | --- | --- | --- | --- | --- | --- | --- |
| **Variables** | **First Dietary Pattern (Fatty Acids)** | | | | **Second Dietary Pattern (Minerals And Vitamins)** | | | | **Third Dietary Pattern (Poly Unsaturated Fatty Acid)** | | | | |
|  | **Q1** | **Q2** | **Q3** | **Q4** | **Q1** | **Q2** | **Q3** | **Q4** | **Q1** | **Q2** | **Q3** | **Q4** | |
| **Total fat g†** | 53.66±0.77 | 66.75±0.76 | 77.97±0.73 | 107.96±1.00 | 68.28±0.57 | 72.19±0.72 | 74.85±0.81 | 91.01±1.02 | 56.89±0.77 | 67.48±0.82 | 77.12±0.91 | 104.76±1.00 | |
| **Total saturated fatty acid, g** | 14.48±0.32 | 20.41±0.29 | 25.74±0.34 | 38.53±0.47 | 20.86±0.22 | 22.70±0.29 | 24.32±0.33 | 31.29±0.50 | 22.65±0.34 | 23.96±0.37 | 24.61±0.43 | 27.96±0.44 | |
| **Total mono unsaturated fatty acid, g** | 19.85±0.36 | 24.88±0.39 | 29.38±0.32 | 41.13±0.45 | 25.83±0.29 | 27.16±0.33 | 27.93±0.38 | 34.32±0.45 | 21.55±0.34 | 25.72±0.38 | 29.14±0.43 | 38.83±0.46 | |
| **Cholesterol, mg** | 92.05±3.83 | 189.49±3.12 | 300.40±3.01 | 576.05±5.78 | 292.70±3.31 | 290.34±4.35 | 275.70±5.72 | 299.25±4.61 | 302.07±3.59 | 298.96±4.14 | 284.27±3.46 | 272.70±5.81 | |
| [**Caffeine, mg**](https://wwwn.cdc.gov/Nchs/Nhanes/2001-2002/DRXTOT_B.htm#DRXTCAFF) | 10.50±5.45 | 98.79±5.90 | 172.08±7.34 | 293.09±14.29 | 166.05±7.29 | 146.73±5.41 | 129.31±6.53 | 132.38±7.46 | 185.46±8.06 | 148.85±5.59 | 129.75±6.37 | 110.42±9.16 | |
| **Saturated fatty acid 4:0 (butanoic), g** | 0.21±0.01 | 0.35±0.00 | 0.48±0.01 | 0.78±0.01 | 0.29±0.01 | 0.38±0.01 | 0.46±0.01 | 0.69±0.02 | 0.52±0.01 | 0.50±0.01 | 0.43±0.01 | 0.37±0.01 | |
| **Saturated fatty acid 6:0 (hexanoic), g** | 0.11±0.00 | 0.18±0.00 | 0.26±0.00 | 0.42±0.01 | 0.16±0.00 | 0.20±0.00 | 0.24±0.00 | 0.36±0.01 | 0.28±0.00 | 0.26±0.00 | 0.23±0.00 | 0.20±0.00 | |
| **Saturated fatty acid 8:0 (octanoic), g** | 0.09±0.00 | 0.15±0.00 | 0.21±0.00 | 0.33±0.00 | 0.14±0.00 | 0.17±0.00 | 0.19±0.00 | 0.28±0.00 | 0.22±0.00 | 0.21±0.00 | 0.18±0.00 | 0.16±0.00 | |
| **Saturated fatty acid 10:0 (decanoic), g** | 0.16±0.00 | 0.28±0.00 | 0.39±0.10 | 0.63±0.01 | 0.25±0.00 | 0.32±0.00 | 0.37±0.00 | 0.53±0.01 | 0.30±0.02 | 0.53±0.02 | 0.72±0.02 | 1.06±0.04 | |
| **Saturated fatty acid 12:0 (dodecanoic), g** | 0.30±0.02 | 0.53±0.02 | 0.72±0.02 | 1.06±0.04 | 0.53±0.02 | 0.60±0.02 | 0.65±0.03 | 0.83±0.03 | 0.74±0.02 | 0.65±0.02 | 0.63±0.03 | 0.58±0.02 | |
| **Saturated fatty acid 14:0 (tetradecanoic), g** | 0.95±0.04 | 1.58±0.03 | 2.13±0.04 | 3.40±0.06 | 1.50±0.03 | 1.76±0.03 | 1.99±0.03 | 2.81±0.06 | 2.22±0.04 | 2.13±0.04 | 1.93±0.05 | 1.78±0.04 | |
| **Saturated fatty acid 16:0 (hexadecanoic), g** | 8.51±0.16 | 11.49±0.16 | 14.25±0.17 | 20.99±0.24 | 11.95±0.12 | 12.75±0.15 | 13.53±0.17 | 17.02±0.25 | 11.93±0.16 | 13.03±0.18 | 13.82±0.23 | 16.47±0.23 | |
| **Saturated fatty acid 18:0 (octadecanoic) , gm** | 3.66±0.08 | 5.23±0.10 | 6.59±0.10 | 9.93±0.11 | 5.50±0.06 | 5.89±0.07 | 6.19±0.10 | 7.83±0.13 | 5.70±0.10 | 6.11±0.10 | 6.34±0.11 | 7.25±0.12 | |
| **Mono unsaturated fatty acid 16:1 (hexadecenoic), g** | 0.73±0.02 | 1.11±0.02 | 1.46±0.02 | 2.29±0.04 | 1.25±0.02 | 1.30±0.02 | 1.36±0.03 | 1.67±0.03 | 1.35±0.02 | 1.40±0.02 | 1.36±0.03 | 1.47±0.04 | |
| **Mono unsaturated fatty acid 18:1 (octadecenoic), g** | 18.69±0.34 | 23.22±0.36 | 27.28±0.31 | 37.89±0.42 | 24.09±0.26 | 25.27±0.31 | 25.95±0.35 | 31.79±0.43 | 19.64±0.31 | 23.72±0.36 | 27.16±0.40 | 36.55±0.42 | |
| **Protein, g** | 65.11±0.71 | 75.36±0.77 | 82.33±1.03 | 100.69±0.78 | 55.33±0.63 | 70.84±0.67 | 83.07±0.72 | 114.25±1.26 | 79.18±0.89 | 79.74±0.69 | 79.56±0.75 | 85.02±1.12 | |
| **Carbohydrate, g** | 277.09±3.75 | 275.43±3.38 | 365.87±3.44 | 272.44±3.72 | 181.33±2.90 | 241.65±3.37 | 286.99±3.33 | 380.87±3.48 | 250.48±3.38 | 266.34±3.15 | 274.05±3.65 | 299.97±3.36 | |
| **Dietary fiber, g** | 19.22±0.30 | 16.99±0.26 | 14.48±0.42 | 12.17±0.32 | 6.93±0.17 | 12.59±0.24 | 17.47±0.31 | 25.86±0.43 | 14.17±0.51 | 15.05±0.31 | 15.74±0.22 | 17.90±0.28 | |
| **Vitamin E, mg** | 8.09±0.14 | 7.88±0.26 | 7.24±0.38 | 7.52±0.16 | 4.65±0.14 | 6.59±0.18 | 8.00±0.16 | 11.49±0.45 | 5.68±0.47 | 6.24±0.16 | 7.54±0.12 | 11.28±0.17 | |
| **Thiamin, mg** | 1.68±0.02 | 1.62±0.02 | 1.51±0.02 | 1.48±0.02 | 0.80±0.01 | 1.28±0.01 | 1.69±0.02 | 2.52±0.03 | 1.51±0.02 | 1.55±0.02 | 1.56±0.01 | 1.68±0.02 | |
| **Riboflavin, mg** | 1.68±0.02 | 1.81±0.02 | 1.85±0.02 | 2.13±0.02 | 0.95±0.02 | 1.50±0.01 | 1.99±0.02 | 3.02±0.04 | 1.97±0.02 | 1.92±0.02 | 1.82±0.02 | 1.75±0.03 | |
| **Niacin, mg** | 21.80±0.30 | 22.49±0.33 | 22.33±0.46 | 22.77±0.27 | 13.89±0.28 | 19.35±0.23 | 23.59±0.35 | 32.56±0.58 | 20.83±0.52 | 21.56±0.27 | 21.86±0.26 | 25.15±0.35 | |
| **Vitamin B6 , mg** | 1.95±0.03 | 1.89±0.02 | 1.74±0.03 | 1.68±0.02 | 0.86±0.02 | 1.47±0.01 | 1.99±0.02 | 2.94±0.04 | 1.81±0.03 | 1.78±0.02 | 1.75±0.02 | 1.92±0.03 | |
| **Total folate, μg** | 448.82±6.47 | 413.80±4.99 | 366.74±5.75 | 334.86±5.91 | 182.56±5.11 | 315.54±4.06 | 429.09±6.28 | 637.03±10.43 | 370.95±6.23 | 379.92±5.85 | 388.87±4.67 | 424.49±4.60 | |
| **Vitamin B12 , µg** | 3.50±0.17 | 4.52±0.19 | 4.97±0.15 | 6.96±0.28 | 2.70±0.13 | 4.24±0.15 | 5.10±0.14 | 7.91±0.34 | 5.88±0.22 | 5.09±0.16 | 4.64±0.19 | 4.34±0.22 | |
| [**Vitamin C, mg**](https://wwwn.cdc.gov/Nchs/Nhanes/2001-2002/DRXTOT_B.htm#DRXTVC) | 129.78±2.63 | 118.08±3.52 | 95.82±3.00 | 69.71±3.48 | 33.32±2.44 | 77.98±2.27 | 118.87±2.75 | 183.21±5.27 | 107.43±3.41 | 102.74±3.18 | 100.52±2.55 | 102.71±3.60 | |
| **Calcium, g** | 724.38±13.43 | 769.70±14.35 | 763.55±25.86 | 832.83±17.21 | 311.05±10.90 | 573.77±10.97 | 828.85±14.09 | 1367.79±34.89 | 848.71±34.06 | 810.40±16.22 | 750.98±15.80 | 680.37±15.26 | |
| **Phosphorus, g** | 1130.12±12.08 | 1217.09±11.05 | 1241.09±13.02 | 1442.97±11.72 | 725.97±9.59 | 1042.83±9.76 | 1321.45±8.66 | 1941.03±21.36 | 1246.40±15.01 | 1251.73±11.64 | 1237.80±11.52 | 1295.34±14.54 | |
| **Magnesium, mg** | 287.81±3.01 | 280.12±2.40 | 262.89±5.66 | 264.56±3.45 | 137.61±2.76 | 222.83±2.21 | 297.25±3.09 | 437.70±7.59 | 266.55±7.02 | 267.47±3.15 | 267.61±2.58 | 293.75±1.86 | |
| **Iron, mg** | 15.89±0.24 | 15.37±0.20 | 14.31±0.35 | 14.21±0.22 | 7.44±0.18 | 12.14±0.13 | 16.13±0.30 | 24.08±0.42 | 14.71±0.48 | 14.70±0.21 | 14.81±0.11 | 15.56±0.20 | |
| [**Zinc, mg**](https://wwwn.cdc.gov/Nchs/Nhanes/2001-2002/DRXTOT_B.htm#DRXTZINC) | 9.94±0.17 | 11.12±0.17 | 11.78±0.33 | 13.63±0.23 | 6.53±0.18 | 9.59±0.18 | 12.10±0.15 | 18.25±0.36 | 12.34±0.36 | 11.57±0.19 | 11.56±0.16 | 11.00±0.25 | |
| **Copper, mg** | 1.36±0.02 | 1.32±0.02 | 1.26±0.03 | 1.31±0.02 | 0.70±0.01 | 1.10±0.01 | 1.40±0.02 | 2.04±0.04 | 1.26±0.04 | 1.25±0.02 | 1.28±0.01 | 1.45±0.02 | |
| **Sodium, mg** | 3045.39±37.92 | 3257.82±37.84 | 3322.32±36.41 | 3755.17±53.14 | 2108.05±28.59 | 2778.02±27.40 | 3480.41±40.09 | 5014.22±47.56 | 3008.92±43.22 | 3270.98±35.98 | 3353.31±35.12 | 3747.50±49.87 | |
| **Potassium, mg** | 2579.78±19.67 | 2658.42±14.78 | 2574.75±20.35 | 2732.31±22.81 | 1318.30±17.64 | 2145.87±12.68 | 2850.77±17.98 | 4230.32±35.57 | 2690.90±25.03 | 2625.72±14.63 | 2576.50±20.82 | 2652.15±22.77 | |
| **Selenium, µg** | 89.05±1.37 | 99.66±1.27 | 108.99±1.13 | 133.21±1.95 | 77.10±1.37 | 96.48±1.31 | 109.14±1.56 | 148.19±1.45 | 100.72±1.23 | 104.42±0.73 | 106.84±1.40 | 118.93±1.85 | |
| **Total poly unsaturated fatty acid , g** | 15.20±0.11 | 15.54±0.14 | 15.51±0.14 | 17.80±0.18 | 15.34±0.11 | 15.72±0.10 | 15.75±0.16 | 17.24±0.18 | 6.46±0.12 | 11.21±0.10 | 16.54±0.10 | 29.84±0.23 | |
| **Mono unsaturated fatty acid 20:1(Eicosenoic), g** | 0.14±0.00 | 0.17±0.00 | 0.19±0.00 | 0.25±0.01 | 0.18±0.00 | 0.18±0.00 | 0.17±0.00 | 0.21±0.01 | 0.11±0.00 | 0.14±0.00 | 0.18±0.00 | 0.32±0.01 |  |
| **Mono unsaturated fatty acid 22:1, g** | 0.03±0.00 | 0.03±0.00 | 0.03±0.00 | 0.06±0.01 | 0.03±0000 | 0.03±0.00 | 0.03±0.00 | 0.07±0.01 | 0.02±0.00 | 0.03±0.00 | 0.03±0.00 | 0.07±0.01 |  |
| **Poly unsaturated fatty acid 18:4, g** | 13.54±0.10 | 13.79±0.13 | 13.67±0.13 | 15.57±0.16 | 13.64±0.10 | 13.87±0.10 | 13.90±0.15 | 15.17±0.16 | 5.49±0.11 | 9.81±0.09 | 14.66±0.08 | 26.63±0.22 |  |
| **Poly unsaturated fatty acid 18:3 (Octadecatrienoic), g** | 1.29±0.02 | 1.33±0.01 | 1.33±0.01 | 1.55±0.02 | 1.25±0.01 | 1.34±0.02 | 1.37±0.02 | 1.54±0.02 | 0.63±0.01 | 0.98±0.01 | 1.40±0.01 | 2.49±0.03 |  |
| **Poly unsaturated fatty acid 18:4, g** | 0.00±0.00 | 0.00±0.00 | 0.00±0.00 | 0.00±0.00 | 0.003±0.00 | 0.005±0.00 | 0.006±0.00 | 0.008±0.00 | 0.003±0.00 | 0.003±0.00 | 0.006±0.00 | 0.01±0.00 |  |
| **Poly unsaturated fatty acid 20:4 (Eicosatetraenoic), g** | 0.07±0.00 | 0.11±0.00 | 0.15±0.00 | 0.24±0.00 | 0.14±0.00 | 0.14±0.00 | 0.14±0.00 | 0.14±0.00 | 0.13±0.00 | 0.14±0.00 | 0.14±0.00 | 0.16±0.00 |  |
| **Poly unsaturated fatty acid 20:5(Eicosapentaenoic), g** | 0.03±0.00 | 0.04±0.00 | 0.04±0.00 | 0.05±0.00 | 0.03±0.00 | 0.04±0.00 | 0.04±0.00 | 0.06±0.00 | 0.03±0.00 | 0.03±0.00 | 0.04±0.00 | 0.07±0.00 |  |
| **Poly unsaturated fatty acid 22:5 (Docosapentaenoic), g** | 0.01±0.00 | 0.01±0.00 | 0.02±0.00 | 0.02±0.00 | 0.01±0.00 | 0.01±0.00 | 0.01±0.00 | 0.02±0.00 | 0.01±0.00 | 0.01±0.00 | 0.01±0.00 | 0.03±0.00 |  |
| **Poly unsaturated fatty acid 22:6 (Docosahexaenoic), g** | 0.07±0.00 | 0.08±0.00 | 0.08±0.00 | 0.10±0.00 | 0.06±0.00 | 0.08±0.00 | 0.08±0.00 | 0.10±0.00 | 0.06±0.00 | 0.06±0.00 | 0.08±0.00 | 0.13±0.00 |  |
| * The highlights indicate the significant contribution of the nutrient as constituent element of the corresponding dietary pattern. †p-values for linear trend across quarters of dietary pattern. | | | | | | | | | | | | |  |
